# Capturing coupled riparian and coastal disturbance from industrial mining using cloud-resilient satellite time series analysis

**DOI:** 10.1038/srep35129

**Published:** 2016-10-11

**Authors:** Michael Alonzo, Jamon Van Den Hoek, Nabil Ahmed

**Affiliations:** 1 Biospheric Sciences Laboratory, NASA’s Goddard Space Flight Center, Greenbelt, MD 20771, USA; 2 Department of Environmental Science, American University, Washington, DC 20016, USA; 3 Geography & Geospatial Science, College of Earth, Ocean, and Atmospheric Sciences, Oregon State University, Corvallis, OR 97331, USA; 4 The Cass School of Architecture, London Metropolitan University, London, UK

## Abstract

The socio-ecological impacts of large scale resource extraction are frequently underreported in underdeveloped regions. The open-pit Grasberg mine in Papua, Indonesia, is one of the world’s largest copper and gold extraction operations. Grasberg mine tailings are discharged into the lowland Ajkwa River deposition area (ADA) leading to forest inundation and degradation of water bodies critical to indigenous peoples. The extent of the changes and temporal linkages with mining activities are difficult to establish given restricted access to the region and persistent cloud cover. Here, we introduce remote sensing methods to “peer through” atmospheric contamination using a dense Landsat time series to simultaneously quantify forest loss and increases in estuarial suspended particulate matter (SPM) concentration. We identified 138 km^2^ of forest loss between 1987 and 2014, an area >42 times larger than the mine itself. Between 1987 and 1998, the rate of disturbance was highly correlated (Pearson’s *r* = 0.96) with mining activity. Following mine expansion and levee construction along the ADA in the mid-1990s, we recorded significantly (*p* < 0.05) higher SPM in the Ajkwa Estuary compared to neighboring estuaries. This research provides a means to quantify multiple modes of ecological damage from mine waste disposal or other disturbance events.

Papua is the easternmost province in the archipelago nation of Indonesia and a global ethno-linguistic and biodiversity hotspot. Home to 1.5 million indigenous peoples who speak over 250 languages^1^, the province also boasts over 17,700 species of flora and fauna, 32 million ha of old growth tropical rainforest and mangroves, and one of the world’s richest marine reef environments with 565 species of coral^2^. Papua’s Grasberg minerals district contains the world’s largest proven gold reserve (28.2 million ounces) and the second largest proven copper reserve (29 billion lbs) as of December 2014^3^. Since 1973, PT Freeport Indonesia (PT-FI), through contract with the Indonesian government, has excavated an open-pit mine (hereafter referred to as “Grasberg”) that produced 18,600 metric tons of ore per day by 1988 and that reached a peak of 237,800 tons per day by 2001^3^. Grasberg is not only one of the most productive gold or copper mines in the world^4^, it is also the largest tax payer in Indonesia^5^ and generates over 50% of provincial gross domestic product as of 2005^6^.

Grasberg sits at 4,270 meters above sea level near the headwaters of the Aghawagon-Otomona-Ajkwa (hereafter, “Ajkwa”) river system that drains an approximately 2,100 km^2^ watershed into the Arafura Sea. Over the last 30 years, Grasberg tailings have been deposited directly into the Ajkwa river system for transport to the Ajkwa Deposition Area (ADA; presently known as the ModADA for “Modified ADA”; Fig. 1). Of the 1.3 billion metric tons of copper ore excavated during our 1987–2014 study period^3^,^7^,^8^, 1–1.5% was viable mineralized copper while 97% was discharged as mine tailings into the Ajkwa river system^9^. With increased discharge and continued heavy metal-rich tailing deposition, the river bed has dramatically aggraded and the channel capacity has been reduced. Mine tailings that do not settle within the ADA are presumed to reach the Arafura Sea–in violation of Indonesian environmental law^10^–where we hypothesize that they increase coastal suspended particulate matter (SPM) and heavy metal concentrations^11^.

Neither the ecological nor socio-economic costs of such intensive and protracted mining have been sufficiently assessed. Since the opening of the mine in 1973, the Amungme people who traditionally lived in the region immediately surrounding Grasberg have been repeatedly displaced by the Indonesian government as the mine has expanded^7^. Downstream towards the coastal lowlands, the Kamoro people have lost traditional staples as the aggrading ADA has inundated Sago palm (*Metroxylon sagu*) and Matoa fruit (*Pometia pinnata*) trees. Traditional fisheries, such as mollusk, on which the Kamoro people depend, have similarly been degraded by elevated SPM and associated copper toxicity^10^,^12^,^13^. Despite prolonged social protest, both groups have had their settlements destroyed, communities frayed, livelihoods imperiled, and have been witness to violence from military, police, and security forces^14^,^15^,^16^,^17^.

After more than three decades of large-scale mining, the impacts of Grasberg tailing disposal on Papua’s forest and estuarial ecosystems have received only minimal study in part because of restricted access to the ADA and regional political instability^13^,^18^,^19^. Repeat remote sensing from satellite platforms, alternatively, allows for monitoring of spatially diffuse and temporally protracted environmental change with reduced need for physical site access. Moderate resolution (30 m) Landsat satellite imagery, in particular, have been successfully used to characterize mining operations and impacted ecosystems^20^,^21^,^22^,^23^. In the ADA, Paull *et al.*^13^ used Landsat imagery to characterize synoptic vegetation changes between 1988, 1996, and 2004, finding extensive human-induced land cover change directly attributable to mining activities via tailings deposition and indirectly via urbanization. This study did not consider changes to coastal conditions and was restricted to only three image dates because of persistent cloud cover and manual change-detection methods; as a result, Paull *et al.*^13^ could not characterize correlation between mine production and downstream environmental consequences.

In this study, we quantified ridgeline-to-coast environmental changes from Grasberg through the Ajkwa Estuary to the Arafura Sea using 199 Landsat satellite images (path 103, row 63) from 1987–2014. We constructed pixel-level land cover histories and sampled coastal water SPM across all image dates, 120 of which have >50% cloud cover. To quantify vegetation disturbance, we developed the Noise Insensitive Trajectory Algorithm (NITA), a novel temporal segmentation algorithm driven, by a robust piecewise orthogonal regression of normalized difference vegetation index (NDVI) time series data that captures both acute and protracted change trajectories and is resilient to atmospheric contamination. We consider a steep annual decrease of >0.10 NDVI/year to be “disturbance” while lower rates are considered “decline”. To characterize changes in coastal water quality, we used a Landsat red band-based indicator of SPM and compared concentrations before and after 1998, the year in which we detected peak vegetation disturbance. Leveraging publicly available annual reports from PT-FI and data collected by WALHI (The Indonesian Forum for the Environment^10^), we show that vegetation disturbance directly correlates with the interannual rate of Grasberg tailings production and, critically, that SPM concentrations in the Ajkwa Estuary have significantly (p < 0.05) increased since 1998, indicating a failure by PT-FI to confine tailings and associated heavy metals to the ADA^24^.

## Results

### River aggradation, vegetation inundation, and coastal deposition

Between 1987 and 2014, 138 km^2^ of rainforest, mangrove, and agricultural lands in our combined ADA/Urban study region experienced substantial vegetation loss (Fig. 2, Supplementary Video V1 showing modeled NDVI change [0–1 scale] from 1987 to 2014) via aggradation and inundation (95.6%), and, to a lesser extent, urbanization (4.4%; Fig. 3). Acute disturbance was the dominant process within the ADA, where the median yearly loss of NDVI was 0.14 and cumulative forest disturbance reached 132 km^2^ by 2006 (Fig. 2). Of this total, 58.2 km^2^ of ADA land experienced either stable or ephemeral vegetation regrowth (i.e., disturbance followed by increasing NDVI above 0.40). Outside of the ADA, only 17% of vegetation loss was driven by acute disturbance, and the median yearly loss of NDVI was 0.04; the protracted decline apparent in non-ADA pixel trajectories is typical of urbanization in this study area (Fig. 3).

Between 1987 and 1997, the aggraded extent of the ADA increased by 71.0 km^2^ (Fig. 2, Supplementary Video V1) at a rate that was highly correlated (Pearson’s *r* = 0.96) with annual mine production as reported by PT-FI (Fig. 4). After 1998, the lateral expansion of the ADA was constrained by levees built by PT-FI; the presence of these levees resulted in a sharp decline in the rate of vegetation loss (Fig. 4). However, ore throughput remained consistently high at >200,000 tons per day from 1998–2007 and >100,000 tons per day through the end of our study in 2014. Given this, we hypothesized there would be a statistically significant increase in tailings deposition southward into the Ajkwa Estuary and the Arafura Sea. To evaluate this hypothesis, we measured median Landsat-derived SPM concentrations at 21 estuarial outlets along the Arafura coast averaging along three-pronged sampling transects extending outwards from the coastline and then compared 90^th^ percentile values between 1987–1997 and 1998–2014 at each outlet (Fig. 5, Supplementary Figure S1). Median SPM values along each transect were used to represent typical values at each outlet and minimize the influence of outlier pixels (e.g., unmasked clouds) while 90^th^ percentile values were used for temporal aggregation in recognition that, particularly in montane systems, most particulate matter is transported during a small number of high-flow days^25^.

As anticipated, SPM measured near the ADA outlet increased from 1998 to 2014 even while 90^th^ percentile SPM concentration at 20 nearby river outlets decreased (Fig. 5). Evaluated with 2 km transects, the 90^th^ percentile SPM concentration at the ADA outlet increased by 16% in the post-1998 study period while non-ADA concentrations in the same period declined by 36% compared to the pre-1998 period. On a per-year basis, the difference in annual SPM values at the ADA vs. non-ADA sites appears minimal or variable prior to 1998 but from 1998 onward, there are significantly higher concentrations (two-sample t-test p < 0.001) in the ADA for each year (Fig. 6).

The relative increase in SPM at the ADA outlet remained significantly higher (two-sample *t*-test p < 0.05) than non-ADA outlets for transects up to 2 km in length from the coastline after which transport, deposition, or dilution in open waters equilibrated ADA outlet SPM concentrations with those of nearby river system outlets. This comparison of SPM at ADA and non-ADA outlets also allowed us to control for the influence of temporally or spatially variable precipitation patterns.

Compared with the Outer Ajkwa River Estuary and the Arafura Sea, the Upper Ajkwa River Estuary at the ADA outlet shows a more dramatic increase in SPM (Fig. 7). With Landsat data we estimate the pre-1998 90^th^ percentile value at 202 g/m^3^ and the post-1998 value at 375 g/m^3^. This latter value is reasonably aligned with year 2000 *in situ* estimates: WALHI reports a median Upper Estuary value of 503 g/m^3^ in 2000 (2006). Though we were unable to directly compare *in situ* SPM values with Landsat-derived SPM due to different measurement dates, our estimates reflect both the substantial increase in Upper Estuary SPM after levee construction in 1998 and the attenuation of SPM from the Ajkwa Estuary to the Arafura Sea due to hydraulic sorting, deposition, and dilution of tailings^11^. Where WALHI reports median SPM concentration of 50 g/m^3^ in the Lower Estuary in 2000 and predicted SPM to reach 200 g/m^3^ by 2014, we estimate a 90^th^ percentile concentration of 293 g/m^3^ (median concentration = 43 g/m^3^) along 1 km transects in 2000 and a 90^th^ percentile concentration of 227 g/m^3^ (median = 55 g/m^3^) in 2014. Here, we report both 90^th^ percentile and median values of all image dates for each year to convey the importance of the specific date of SPM measurement in characterizing this highly dynamic fluvial system (e.g., Fig. 6).

It is possible that, in some image dates, the presence of silt may bias SPM results. However, we believe this to be a rare occurrence because red reflectance values of >17% are essentially disallowed by the SPM model (i.e., the result becomes negative). Furthermore, using an empirically-based red band model, Lobo *et al.* (2015) found that a red-band reflectance model for total suspended solids was tractable for reflectance values up to 22%, and likely up to 35%, which equates to ~300 g/m^3^.

### Validation of remote sensing results

This study is driven by remote sensing time series analysis because systematically acquired ground data appropriate for measuring the impacts of mine-waste disposal on both riverine and estuarial ecosystems are extremely difficult to obtain in this remote and restricted environment. Landsat-derived results cannot replace *in situ* measurements and we acknowledge that the lack of contemporaneously acquired ground data contributes to the uncertainty of our results. However, with respect to extreme events leading to landscape denudation, the use of Landsat time series has been shown to be sufficient in the absence of field data^23^,^26^. Moreover, first-order estimates of environmental degradation have great value in underdeveloped or politically unstable areas where ecological damage may otherwise go undocumented^27^,^28^.

Lacking *in situ* data, NITA trajectories based on NDVI time series data were validated against 100 manually delineated trajectories representative of all key strata present in this study (e.g., disturbance dates and types, as well as stable forest; Supplementary Figure S2). NDVI time series values were simulated 100 times for each trajectory based on sets of valid image dates sampled directly from the data (i.e., accounting for spatial and temporal autocorrelation of cloud cover) and a data-driven noise model yielding 10,000 accuracy assessment “pixels” with known trajectories and breakpoints. Our selected input parameter set (see Methods section) resulted in mean overall error in number of trajectory segments of 0.54, mean error (for pixels with disturbance) of 147 days in estimating the date-before-disturbance breakpoint, mean error of 83 days for estimating the date-of-nadir breakpoint, and mean root mean squared error (RMSE) of 0.02 NDVI units compared to manual trajectories (Supplementary Figure S3).

The most common trajectory observed in this study was the three-segment pixel history representing a single inundation event with no vegetation recovery (Fig. 3a). For this history, the mean error (n = 3500 simulated pixels for disturbance dates between 1990 and 2006) in number of segments was 0.75, error in the date-before-disturbance breakpoint was 124 days, date-of-nadir breakpoint was 105 days, and mean RMSE was 0.03. Generally, due to data gaps from cloud cover, date-before disturbance was underestimated and date-of-nadir was overestimated. Regarding parameter sensitivity, there was a consistent tradeoff between parameterizations that yielded high accuracy with respect to number of segments compared to those that yielded more precise fits (e.g., lower RMSE and lower error in disturbance dating).

We also compared our estimates of disturbance extent in 1996 and 2004 to those produced by Paull *et al.*^13^. In 1996 and 2004, we measured 50.2 km^2^ and 131 km^2^ of disturbed vegetation within the ADA, respectively, both of which are less than the approximately 65 km^2^ and 164 km^2^ of disturbance measured by Paull *et al.* The differences between our estimates and those by Paull *et al.* are a product of, first, Paull *et al.* classifying all river pixels as “disturbed” even if a given river pixel was identified as “water” prior to 1987, and, second, different definitions of “disturbance” in the Upper Ajkwa Estuary: Paull *et al.* labeled all pixels within the Upper Estuary as “disturbed” while we more specifically identify disturbance only when a given pixel’s NDVI declines below the 0.40 NDVI threshold.

Contemporaneous field validation of SPM measurements was not possible due to lack of site access. However, for purposes of illustration, we present our Landsat-derived SPM values with *in situ* measurements taken in previous field campaigns throughout the Ajkwa Estuary and reported by WALHI as well as sediment transport model outputs from PT-FI’s Environmental Risk Assessment (ERA) conducted in 2000^10^.

## Discussion

### Cascading effects of Grasberg tailings deposition

We used a single dataset (199 Landsat surface reflectance images) to document coupled riparian vegetative disturbance and coastal water quality degradation, and to examine relationships between these changes and Grasberg tailings deposition. The cloud-resilient disturbance detection approach developed for this study characterizes the pattern of disturbance and degradation in a manner that is unprecedented, regionally, with respect to its fine spatial and temporal resolution. Nonetheless, this study builds on and contributes to decades of research and advocacy by Papuan environmentalists by illuminating the broad-scale and long-term processes of copper and gold extraction at Grasberg that mutually affect regional ecosystems and indigenous communities.

Indonesia is home to 12.8% of all humid tropical forest clearing^29^, and has outpaced Brazil in this regard since 2012^30^. Clearing has long been driven by economic concerns, most notably the expansion of oil palm plantations during the 1990s^31^. Within Indonesia, the Sumatra and Kalimantan lowland forests have borne the brunt of old-growth forest clearing leaving the relatively isolated Papua with some of the largest expanses of intact lowland forest^31^. It is perhaps this geographic isolation, alongside increasing global demand for cheap copper that gave rise to the regulatory environment in which mining operations and waste disposal take priority over regional deforestation and associated human impacts. Indeed, during the study period, Grasberg milled over 1.3 billion tons of ore, among the top global producers in terms of throughput.

Given the relatively low natural sediment carrying capacity of the Ajkwa river system (estimated to be between 15,000 and 20,000 tons per day^7^), the added throughput of approximately 19,000 tons per day of Grasberg tailings during the late 1980’s and early 1990’s pushed the river system beyond its capacity. The tailings volume coupled with a build-up of log debris in 1990 led to overland sheeting and intrusion of contaminated water into the neighboring Minajerwi and Kopi rivers (Supplementary Video V1, 1989–1994 date range; Fig. 2c,d). This flooding, alongside a report highlighting human rights violations by the Indonesian armed forces near Grasberg, prompted PT-FI to begin constructing a 50 km long levee on the west banks of the Ajkwa River in 1994 to protect Timika, the rapidly urbanizing city of residence for many Grasberg employees^9^,^32^ (Fig. 2a). Since the Ajkwa could not expand beyond the western levee, it spread eastward into an area that, according to PT-FI, was approved *ex post facto* by several Indonesian government ministries under President Suharto as a legal expansion of the ADA^9^. By 1995, an eastern levee (eventually reaching 35 km in length) had been partially constructed (Fig. 2b) and, by 1997, the channelized river then receiving ~125,000 tons per day of tailings surged 16 km southward (Supplementary Video V1, date range 1996–1999) into the Ajkwa Estuary. As of 2001, it was reported that 93% of the total river sediment load (~258,000 TPD) at the Otomona Bridge was mine derived^33^. While our remote sensing analysis focused on ADA disturbance between 1987 and 2014, there is clear evidence of more recent forest inundation and degradation in the south, hastened by western levee expansion between 2012 and 2016 (Supplementary Figure S4).

We hypothesized that SPM concentrations in the Arafura Sea adjoining the Ajkwa Estuary would increase significantly after 1998, the year of peak ADA expansion, after which vegetation disturbance rates declined following eastern and western levee completion. We measured SPM using a red band reflectance metric that, while not tuned empirically to local water bodies, has been successfully used across various geographies^34^,^35^. At 20 non-ADA outlets, SPM concentrations decreased after 1998. Independent of these trends however, we found, in correspondence with our hypothesis, that SPM concentrations within 2 km of the ADA outlet were higher after 1998 (Fig. 5). Measurement of SPM is difficult with relatively infrequent cloud-free pixels due to the extremely high variability in SPM discharge. We illustrate the principle that a small number of days deliver a high percentage of SPM in Fig. 6. Here, even with limited sampling, the upwardly biased interquartile range shows that while the “typical daily” (i.e., median) SPM content may be in the range of 50 g/m^3^, there are numerous dates where this value is much higher.

The high level of suspended solids transported into the Arafura Sea correlates with increased concentrations of dissolved copper. PT-FI’s own *in situ* measurements from 1990 indicated that dissolved copper was still 4 times higher than background levels at 10 km into the Arafura Sea^10^. While these estimates have large uncertainties, it is clear that SPM concentrations since 1998 have consistently been above 40 g/m^3^, a level of contamination that has been shown to directly contribute to mortality in aquatic plants and affect the reproductive cycle of invertebrates and fish^36^. The photosynthetic capacity of macrophytes such as phytoplankton is reduced at SPM concentrations over 10 g/m^3^ and severely hindered at concentrations greater than 40 g/m^3^ 37; indeed, Australian guidelines for tropical lowland rivers and estuaries suggest an upper bound of 20 g/m^3^ for maintenance of healthy aquatic communities^38^.

### Socio-environmental consequences and industrial transparency in Papua

In 1991, the Indonesian government acquired part ownership of PT-FI^7^ and made concrete the conflict of interest between environmental regulation enforcement and mining operations that had been presumed given PT-FI’s status as the nation’s largest tax payer^17^,^39^. A 2001 mandate by the Indonesian government requiring that PT-FI build a containment dam in the lower ADA to prevent tailings from reaching the Ajkwa Estuary was never enforced and later modified such that only 75% of total suspended solids that enter the ADA are required to remain within the ADA^10^. By 2006, PT-FI had already discharged more than 1 billion tons of tailings into the Ajkwa river system in direct contravention to prohibitions on riverine disposal of mine waste stipulated in the Indonesian Water Quality Management and Water Pollution Control regulations^10^. These heavy metal-laden tailings will likely remain in the ADA alluvial sediment for centuries after mining has completed^11^,^40^.

Just as Grasberg’s environmental legacy has been inscribed in sheet after deposited sheet in the ADA over the last three decades, so too have PT-FI and its mining operations been inscribed on indigenous Papuan welfare and livelihoods since Grasberg’s opening^14^,^41^. Indigenous Papuans have lost their forest, sago, and, in many ways, the Aghawagon-Otomona-Ajkwa River system, itself, with the ADA’s expansion. An estimated 1.8 million non-Papuans have migrated to the region since 1971^42^. Drawn by employment with PT-FI, the migrant influx has decreased the portion of indigenous Papuans living within the ADA from over 95% in 1967 to less than 15% by 1997^13^. While indigenous Papuans experience ever greater economic and political marginalization^43^, PT-FI has never been obligated to compensate those displaced by mining operations^7^.

This study quantifies and visualizes^44^,^45^ protracted and widespread environmental degradation from industrial mining tailings deposition that has thus far gone undocumented. However, with the 2–3 million year old ore^33^ in Grasberg nearly exhausted, PT-FI has begun transitioning to an entirely underground excavation of nine proven ore bodies^43^ that will be exploited until 2041, the projected date of mine depletion^15^. With the pending shift belowground and out-of-sight, the need for monitoring downstream environmental impacts of Grasberg operations will only increase^13^. With this in mind, we hope that our results provide a measure of accountability regarding extant environmental degradation in Papua and, further, support marginalized indigenous Papuan communities that seek industrial transparency.

## Methods

### Study region

**Figure 1:**
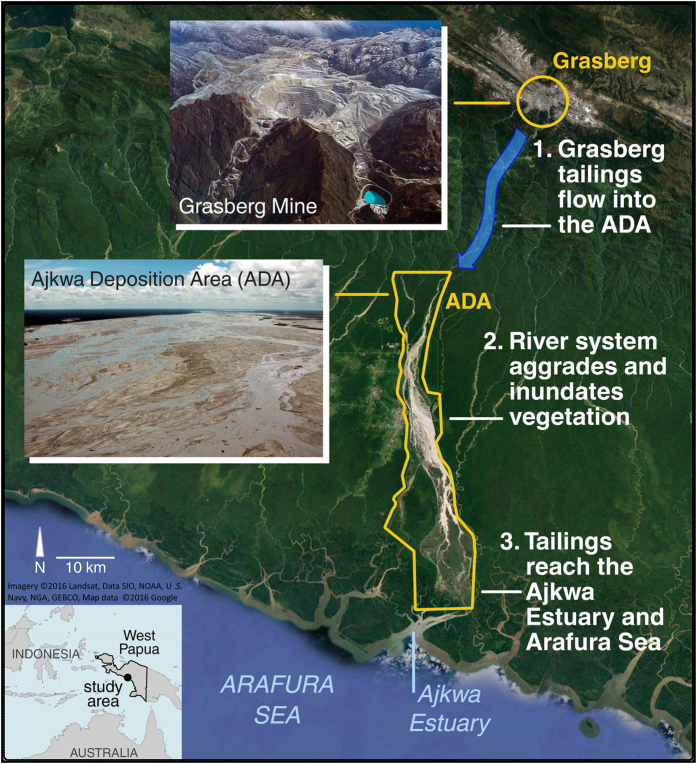
Study area locator map and overview of tailings flow. The Ajkwa Deposition Area (ADA) is bounded by the area potentially disturbed by mine tailings flow. Map created in QuantumGIS 2.12 (www.qgis.org) with cartographic/figure finishing for all figures conducted in Inkscape 0.91 (www.inkscape.org). Photos from Google Earth used with permission.

**Figure 2:**
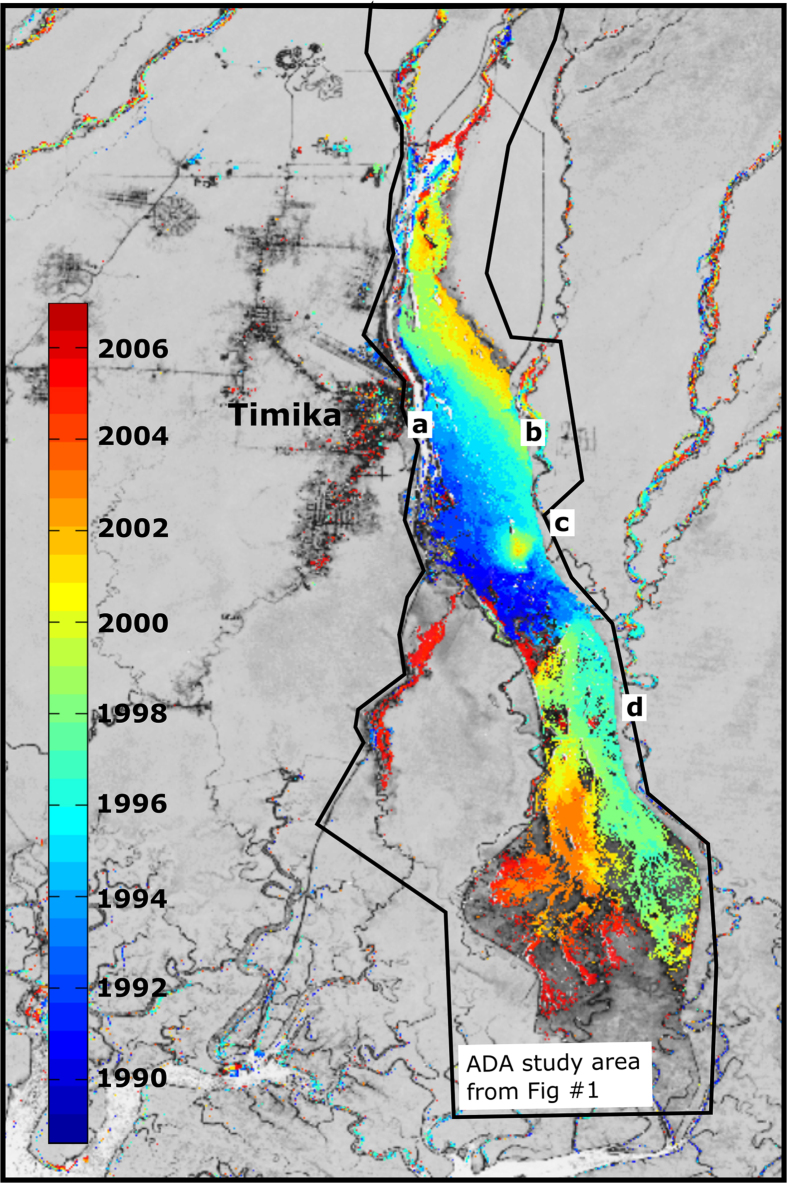
Date of disturbance . Reference points: (**a**) western levee near Timika, (**b**) eastern levee, (**c**) Kopi River, (**d**) Minajerwi River. In this study we recorded no disturbance after 2006, but there was additional southward expansion of the ADA particularly from 2012-2016 (Supplementary Figure S4). Map created in Matlab 2015 (www.mathworks.com) using surface reflectance-corrected Landsat imagery available from the U.S. Geological Survey.

**Figure 3:**
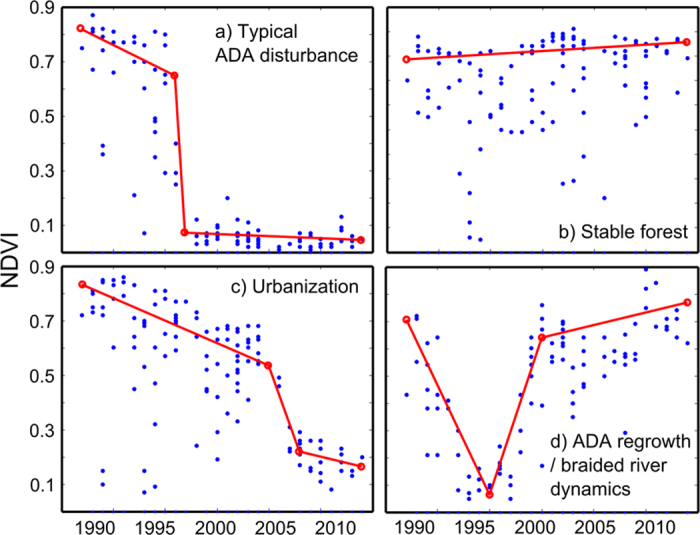
Four representative pixel trajectories: (**a**) typical ADA disturbance showing an acute decrease in NDVI associated with inundation; (**b**) simple, linear trend common for intact forest; (**c**) protracted NDVI decline typically associated with urbanization; (**d**) ephemeral vegetation disturbance and regrowth in braided river system. Blue dots are NDVI values from individual image dates; very low NDVI values may result from cloud cover. Red dots are breakpoint dates outputted from NITA, with segments indicative of disturbance or regrowth.

**Figure 4:**
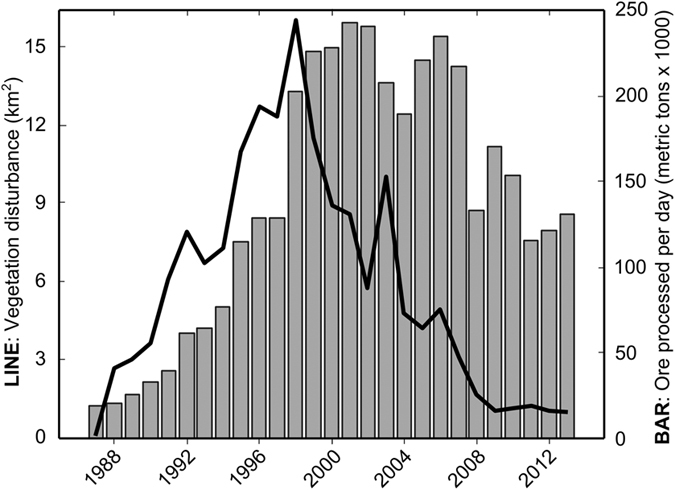
Vegetation disturbance in the ADA (line) overlaid on annual Grasberg ore production (bar). The peak annual disturbance rate is in 1998. Sources for ore processing data are Leith (2003), Mealey (1996), and PT-FI investor reports publicly available online^3^.

**Figure 5:**
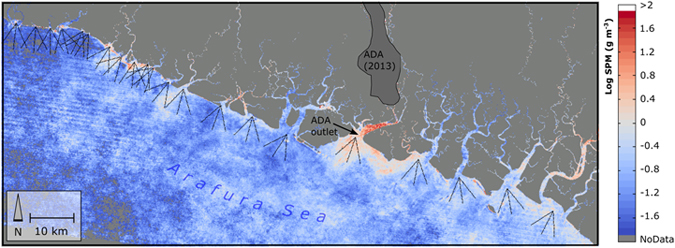
Change in 90^th^ percentile suspended particulate matter (SPM) concentration (log scale) from the pre-1998 period to the 1998–2014 period. Also shown are 21, trident-shaped sampling transects at river outlets on either side of the ADA outlet (shown here: 6 km transects for visual clarity). Each trident contains three transects, each with 200 sample points. Map created in Matlab 2015 (www.mathworks.com) using Landsat surface reflectance imagery available from the U.S. Geological Survey.

Our analysis is set within the Mimika Regency of the Papua province of Indonesia (Fig. 1), downstream from Grasberg, a large-scale, open-pit copper-gold mine. This region includes forested and mangrove areas along the Ajkwa River as well as coastal waters of the Arafura Sea approximately 60 km south of Grasberg. Our study’s first component introduces the Noise Insensitive Trajectory Algorithm (NITA) to examine short- and long-term vegetation disturbance along the river; the second component addresses the spatial diffusion of suspended sediments into Arafura coastal waters. All relevant geographic features fit within a single Landsat tile (WRS-2, Path 103, Row 63).

### Vegetation disturbance using Landsat time series

A remote sensing time series-based approach that was robust to consistent cloud cover and sensitive to spatially diffuse and temporally protracted changes was required to examine the relationship between Grasberg tailings production and regional environmental disturbance. NITA was inspired by significant recent work dedicated to constructing per-pixel land-cover histories using Landsat time series^46^,^47^,^48^,^49^,^50^,^51^,^52^,^53^,^54^. The strengths of these methods range from parsimonious characterization of annual trends^46^,^47^,^48^, to statistical determination of disturbance dates using data driven approaches^49^,^54^, to simultaneous monitoring of seasonal and trend change^50^,^52^, to flexible parameterization based on user needs^51^. However, with some exceptions^52^,^55^, most of the aforementioned algorithms rely on consistent data availability due to relatively low cloud cover. For our study, we sought a disturbance detection algorithm that has ease of implementation, flexible parameterization, the ability to overcome limitations imposed by missing image dates and remnant atmospheric contamination, and which did not require information on sub-annual phenology. Lacking an exhaustive comparison between NITA and existing disturbance detection algorithms, we do not make the assertion that other algorithms could not perform equally well.

We used 199 Landsat 4/5 TM and Landsat 7 ETM + scenes to form a 28-year time series from 1987–2014. Surface reflectance images generated using the Landsat Ecosystem Disturbance Adaptive Processing System (LEDAPS)^56^ were downloaded directly, along with the *fmask* cloud mask product^57^ from the United States Geological Survey’s EarthExplorer website (earthexplorer.usgs.gov). Given the 55% mean scene cloud cover, we developed a vegetation disturbance detection algorithm suitable for high frequency observations that is insensitive to missing data due to atmospheric contamination or Landsat 7’s Scan-Line Corrector data gaps. We performed standard preprocessing including co-registration, spatial subsetting, and cloud masking, and then calculated NDVI^58^ using a single automated process. Though NDVI saturates at high values, it is sensitive to change in photosynthetic biomass below 0.70, typical of our study area^59^.

The NITA algorithm is implemented on each image pixel and uses all image dates containing valid pixel-level spectral information. The inputs into the algorithm are the set of valid image dates (“x”), the accompanying set of spectral index values (“y”), and the user-defined parameters, *max_segment, bail_thresh*, *prctile*, *filt_dist*, and *penalty* each described in Table 1.

An overview of the NITA algorithm as implemented on a single pixel time series follows (also see Fig. 8):

1. Calculate the *noise* variable for the entire times series using the median forward finite difference of the set of spectral values. This variable internalizes atmospheric contamination, geometric errors, and phenology.
2. Establish a single-segment, linear fit in accordance with the *prctile* parameter.
3. Compare the value of *bail_thresh* with the ratio of error of the initial linear fit to *noise*. If *bail_thresh* is not exceeded (e.g., the case of low error and/or high noise), adopt a single-segment model; otherwise, continue.
4. Initiate *NITA build* subroutine and add successive breakpoints until the number of breakpoints reaches *max_segments*. Breakpoints are added at the date (x value) of maximum orthogonal error of the previous fit, after filtering error by *filt_dist*. The y-value of the breakpoint is calculated from the set of spectral values within *filt_dist* image dates of the date of maximum error. If, for example, *prctile* is 75, then the y-value is the approximate 75^th^ percentile value of the *filt_dist* subset of points (Fig. 8).
5. Run the *NITA subtract* subroutine to iteratively remove superfluous breakpoints. The final set of breakpoints minimizes the Bayesian Information Criterion^51^ based on log-likelihood of a lognormal distribution (given all orthogonal distances >0) and the *penalty* parameter. The BIC equation is formulated as:

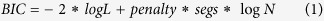


where log*L* is the log-likelihood function value, *penalty* is the user-defined multiplier, *segs* is the number of model segments, and *N* is the number of observations.

The generation of trajectories is based on iterative fits of the piecewise function while the ultimate number of segments is determined with a BIC criterion. Thus, there is no explicit judgement as to what does or does not constitute a “disturbance”. As in *Landtrendr*, the user can decide on the magnitude of change in spectral index values over a given duration should be considered a disturbance^48^. In our study, based on typical forested NDVI values of above 0.8, we consider a disturbance as an event that changes NDVI from above 0.70 to below 0.40 at a rate of >0.10 per year. This disturbance rate reflects rapid NDVI changes in the ADA due to inundation while excluding more protracted NDVI decline associated with degradation in the urbanizing region surrounding Timika. As in Paull *et al.*^13^, we separate regions of urbanization from inundation using the ADA’s western levee^13^ as a physically meaningful dividing line.

### Parameter sensitivity and accuracy assessment through simulation

In order to select the most effective input parameter set, we tested the sensitivity of results against simulated data representing the key pixel trajectories in our Papua study area. Idealized trajectories were manually delineated for disturbances in 1991, 1997, and 2003 as well as braided river dynamics, stable forest, and urbanization processes. For each idealized trajectory, noise was added in two ways: (1) Real sets of valid dates (i.e., cloud- and SLC error-free dates) were generated from 100 pixels randomly sampled throughout the image. For each pixel history, 20 valid date sets were randomly selected from the 100 pixel sample. (2) For each trajectory and each date set, 20 sets of random noise were added according to a non-parametric noise model which, itself, was based on the spectral index distributions of 100 forested pixels. This effectively created 400 example pixels for each of the manually-delineated trajectories in question.

For each trajectory type, *bail_thresh, filt_dist,* and *penalty* were varied to determine their impact on estimates of model complexity (number of segments), fit RMSE compared to the originating trajectory, and, when applicable, date-before-disturbance breakpoint and date-of-nadir breakpoint. We hypothesized that, in particular, changes in *filt_dist* and *penalty* would simultaneously improve accuracy of model complexity while reducing accuracy of disturbance dating and vice versa. The parameters *max_segment* and *prctile* were not tested because their sensitivities can be understood logically; they are provided to the user for fine tuning.

Following selection of a reasonable parameter set (*prctile* = 90, *filt_dist* = 3, *bail_thresh* = 2, *penalty* = 4), we conducted an accuracy assessment using 100 manually-generated trajectories based on real data from 100 pixels with 1 to 5 “true” segments. In the same manner as the parameter testing above, these 100 idealized trajectories were used to simulate missing dates and noise. We simulated 10 sets of missing dates and 10 sets of random noise for each trajectory yielding 3900 “pixels” with one segment, 1100 with 2, 3500 with 3, 1100 with 4, and 400 with 5. These trajectories represented the spectrum of disturbance dates and types present in our study area. Noise standard deviation was set to 0.2 NDVI units, a level commonly found in this study area.

### Spatio-temporal dynamics of coastal suspended particulate matter (SPM)

While larger particulates tend to settle upstream, closer to the mine, smaller-sized particulates may settle along the length of the ADA and even infiltrate coastal waters^9^,^11^. We hypothesized that the transport of tailings through the Ajkwa River and the ADA would increase after 1998 and yield a greater SPM concentration in the Arafura Sea at the river’s outlet. To test this hypothesis, we calculated changes in Landsat-derived SPM concentration (g/m^3^) using the following equation^34^:


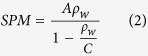


where 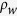
is the water-leaving red band reflectance and *A* (327.84 g/m^3^) and *C* (0.1708) are empirical coefficients specific to the 660 nm center of Landsat TM and ETM+’s red band; variation in 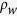 can be attributed to optical characteristics of water rather than atmospheric or glint effect^60^.

To assess changes in SPM concentration before and after 1998, we generated a 199-date SPM time series in the same manner as the NDVI time series discussed above. We sampled SPM concentrations for all image dates along three-pronged, trident-shaped transects, ranging from 1 to 15 km in length, originating from the Ajkwa Estuary directly south of the ADA, which receives the vast majority of Grasberg tailings. In addition, we constructed similar transects (Supplementary Figure S1) at 20 river outlets outside of the ADA to capture baseline changes in SPM that are not likely to be related to mining activity. At the ADA outlet, the SPM concentration for each image date was measured as the median SPM at all sample points along a transect of a given length. The SPM at non-ADA outlets is characterized by the median SPM across all 20 non-ADA transects of a given length for each image date. Spatial averaging using median accounts for the lognormal frequency distribution of SPM values and minimizes the impact of outlier values (frequently from unmasked cloud edges). All *temporal* aggregation was completed using 90^th^ percentile values based on well-established theory that in mountain rivers, half of the annual suspended sediment flux will be transported in <5% of the days^25^. For the purposes of this study, 90^th^ percentile SPM values at the ADA outlet were compared to 90^th^ percentile values at non-ADA outlets for pre-1998 and post-1998 periods. This comparison supports identifying changes unique to the ADA outlet transect by decoupling spatial and temporal variability in SPM from changes in river channel morphology, runoff potential, and variability in rainfall or other mesoscale phenomena that may affect SPM transport, deposition, or dilution.

**Figure 6:**
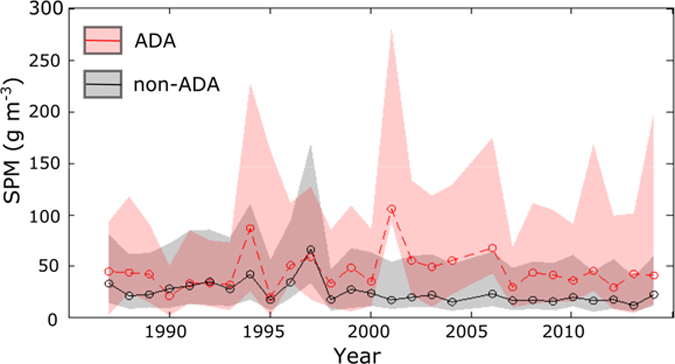
Yearly median SPM concentrations as sampled at 201 transect points (within 2 km-long transects) at the Ajkwa Estuary (the outlet for the ADA) and 4,020 points at 20 other nearby river outlets; shaded regions indicate the variability (interquartile range) of SPM for a given year. This variability results from spatial variation over 201 or 4,020 sample points as well as the 1 to 9 image dates used to calculate the annual, aggregated SPM estimate.

**Figure 7:**
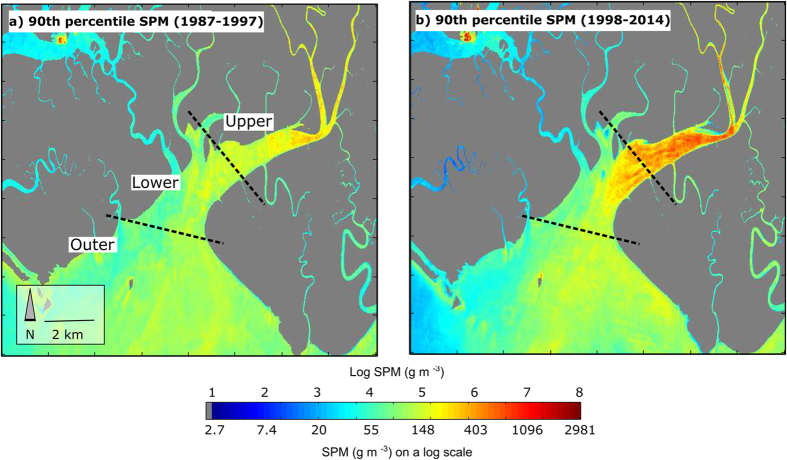
90^th^ percentile SPM concentrations (log scale) in the Upper, Lower, and Outer Ajkwa Estuaries (ADA outlet) from (**a**) 1987 through 1997, and (**b**) 1998 through 2014. Given that spatial averaging was not applied in this map, the highest values may include cloud edges or silt. Map created in Matlab 2015 (www.mathworks.com) using Landsat surface reflectance imagery available from the U.S. Geological Survey.

**Figure 8:**
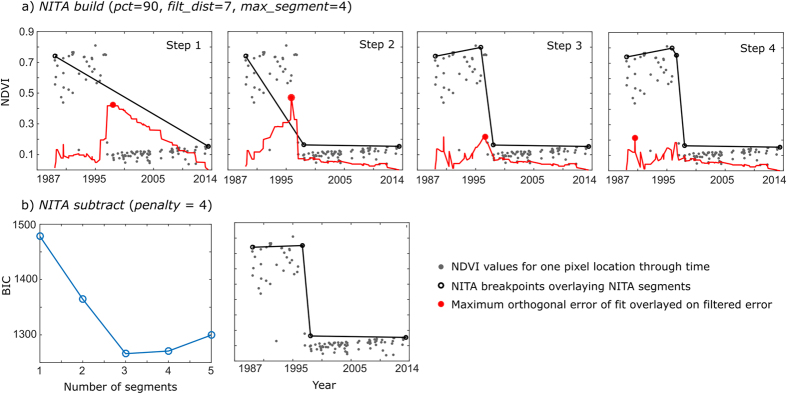
Fitting trajectories using NITA. (**a**) *NITA build* subroutine for “single inundation event” trajectory. Breakpoints are added at each step at the point of maximum, filtered, orthogonal error until *max_segment* is reached. (**b**) *NITA subtract* subroutine. Each breakpoint is iteratively removed and Bayesian Information Criterion (BIC) is calculated. The final piecewise fit minimizes the BIC.

To further explore the effects of high spatial and temporal variability in this system, we use 2 km transects as a case study (Fig. 6) to show the interquartile range of SPM values over space (i.e., across sample transects) and time (i.e., image dates within a single year). This variation in Landsat-derived SPM within a given year results from the high temporal variability in sediment transport and highlights the need to augment single-date point sampling methods through spatially and temporally extensive sampling. It is possible that Landsat’s nominal 16-day revisit period is too infrequent for representative sampling in locations where the majority of the river’s sediment transport occurs over a small minority of days (e.g., rivers predominately fed by rainfall or snow and glacier melt rather than groundwater or lakes^25^).

## Additional Information

**How to cite this article**: Alonzo, M. *et al.* Capturing coupled riparian and coastal disturbance from industrial mining using cloud-resilient satellite time series analysis. *Sci. Rep.* **6**, 35129; doi: 10.1038/srep35129 (2016).

## Supplementary Material

Supplementary Information

Supplementary Video 1

## Figures and Tables

**Table 1:** User defined input parameters for NITA.

Input parameter	Explanation	Default
*prctile*	Inspired by quantile regression. Allows user to determine whether to fit median or other quantiles of data. Particularly useful when spectral index noise is not normally distributed.	50
*bail_thresh*	Only add breakpoints to single-segment trajectory if the ratio of error of the initial linear fit to time series noise is greater than *bail_thresh* value. Improves computational efficiency and reduces likelihood of over-fitting noise.	1
*max_segment*	Maximum number of segments added in *NITA build* subroutine.	10
*filt_dist*	Temporal kernel over which a 1-D median filter is applied to each fit’s error in NITA build subroutine (Fig. 8). Higher values will reduce sensitivity to noise but also to acute disturbances and recoveries.	3
*penalty*	Multiplier to control severity of the Bayesian Information Criterion (BIC; Eq. 1). Higher values result in prioritization of model simplicity (i.e., fewer segments).	1
