# Supplementary material for: Capturing coupled riparian and coastal disturbance from industrial mining using cloud-resilient satellite time series analysis
Source: Sci Rep. 2016 Oct 11;6:35129. doi: 10.1038/srep35129 (PMC5057129; doi:10.1038/srep35129)
Supplement: Supplementary Information [file srep35129-s1.pdf]

Supplementary Materials: Capturing coupled riparian and coastal disturbance from industrial mining using cloud-resilient satellite time series analysis

Authors: Michael Alonzo, Jamon Van Den Hoek, Nabil Ahmed

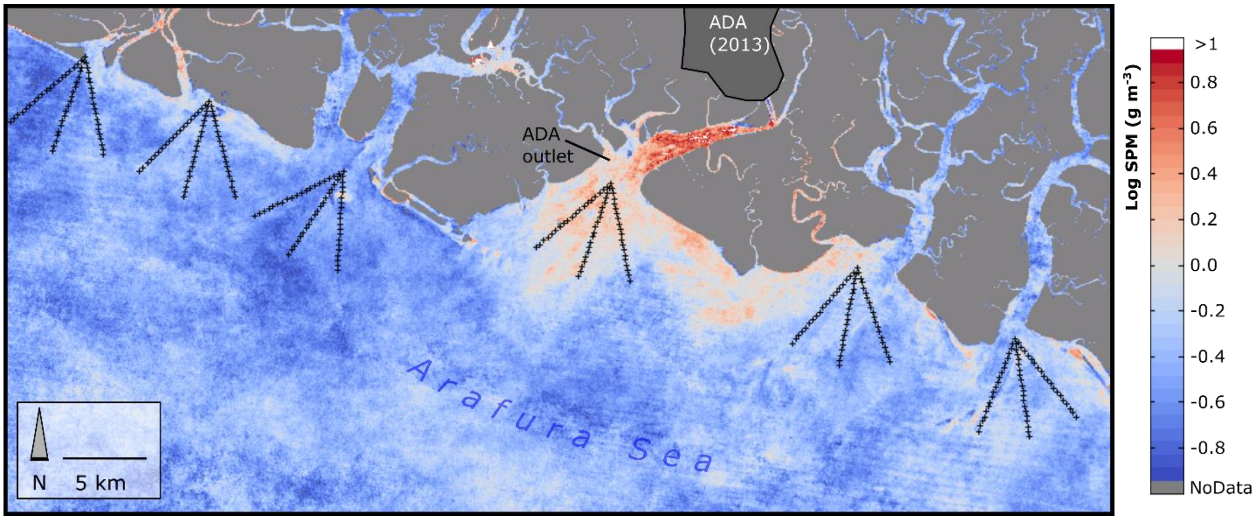

Figure S1: Detail of the "trident" sampling scheme showing 6 tridents at river outlets including the ADA outlet; the full set of tridents is shown in **Figure 5** in the main text. For visual clarity, only one in every ten sample locations is shown per transect. To decouple changes in SPM from rainfall variability or other mesoscale environmental processes, we sample each image date using trident-shaped transects positioned at 20 non-ADA river outlets as well as the ADA's outlet. The trident design allows us to 1) capture dispersion of sediment as it is transported to the Arafura Sea or along the coastline, and 2) measure the distance at which SPM levels at the ADA outlet significantly vary from non-ADA SPM levels. Spatial and temporal averaging were required to reduce the influence of outlier values (e.g., bottom effects and atmospheric contamination) and to better capture long-term trends in a highly variable system (Lobo et al., 2015). Map created in Matlab 2015 (www.mathworks.com) using Landsat surface reflectance imagery available from the U.S. Geological Survey.

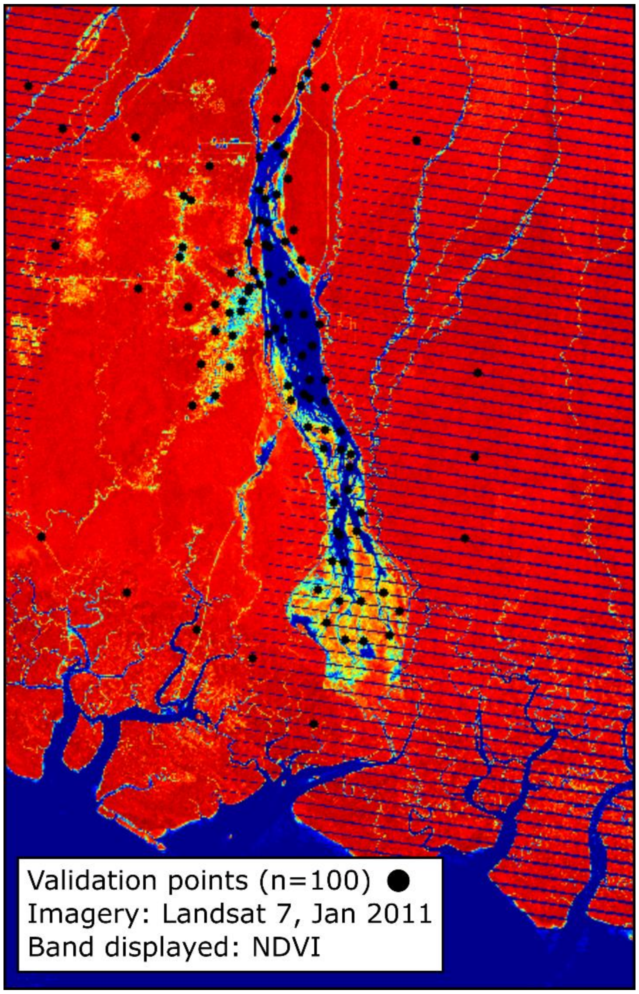

Figure S2: Locations of the 100 validation points for evaluating land cover change (see **Figure S3** below). Map created in Matlab 2015 (www.mathworks.com) using Landsat surface reflectance imagery available from the U.S. Geological Survey.

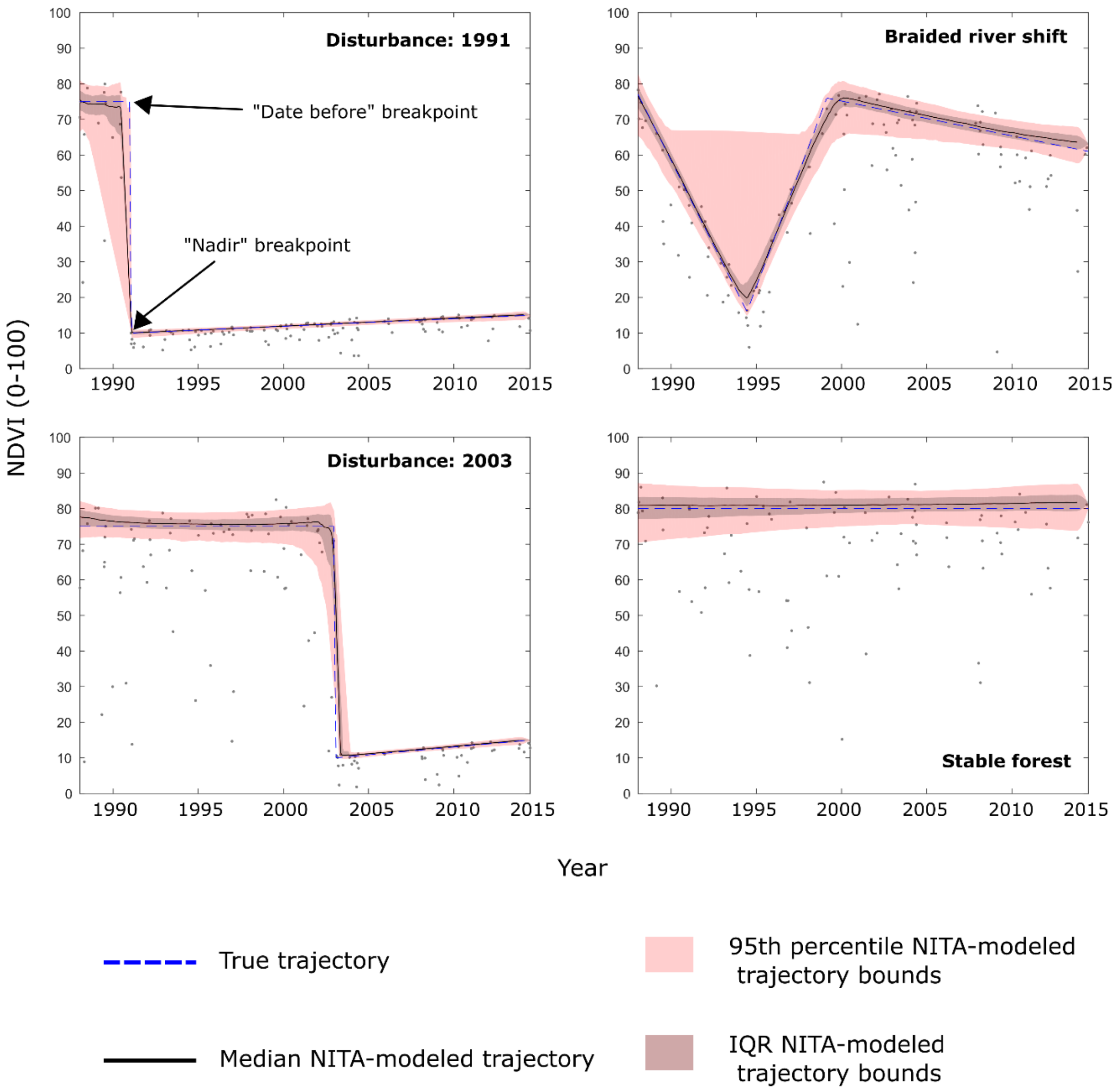

Figure S3: Four examples of the trajectory validation process. For each of 100 manually delineated trajectories, NDVI values were simulated 100 times with varying sets of valid image dates and noise. This yielded an ultimate validation dataset with 10,000 simulated “pixels”. Each of the trajectories displayed in this figure is illustrated with a single instantiation of NDVI values. The median trajectory and the confidence intervals are based on 100 instantiations of the simulation and 100 NITA runs. Accuracy was determined by comparing the “true” trajectories with the NITA-modeled trajectories based on: 1) number of segments, 2) dates of disturbance breakpoints (date-before and date-of-nadir), and 3) root mean squared error of NDVI.

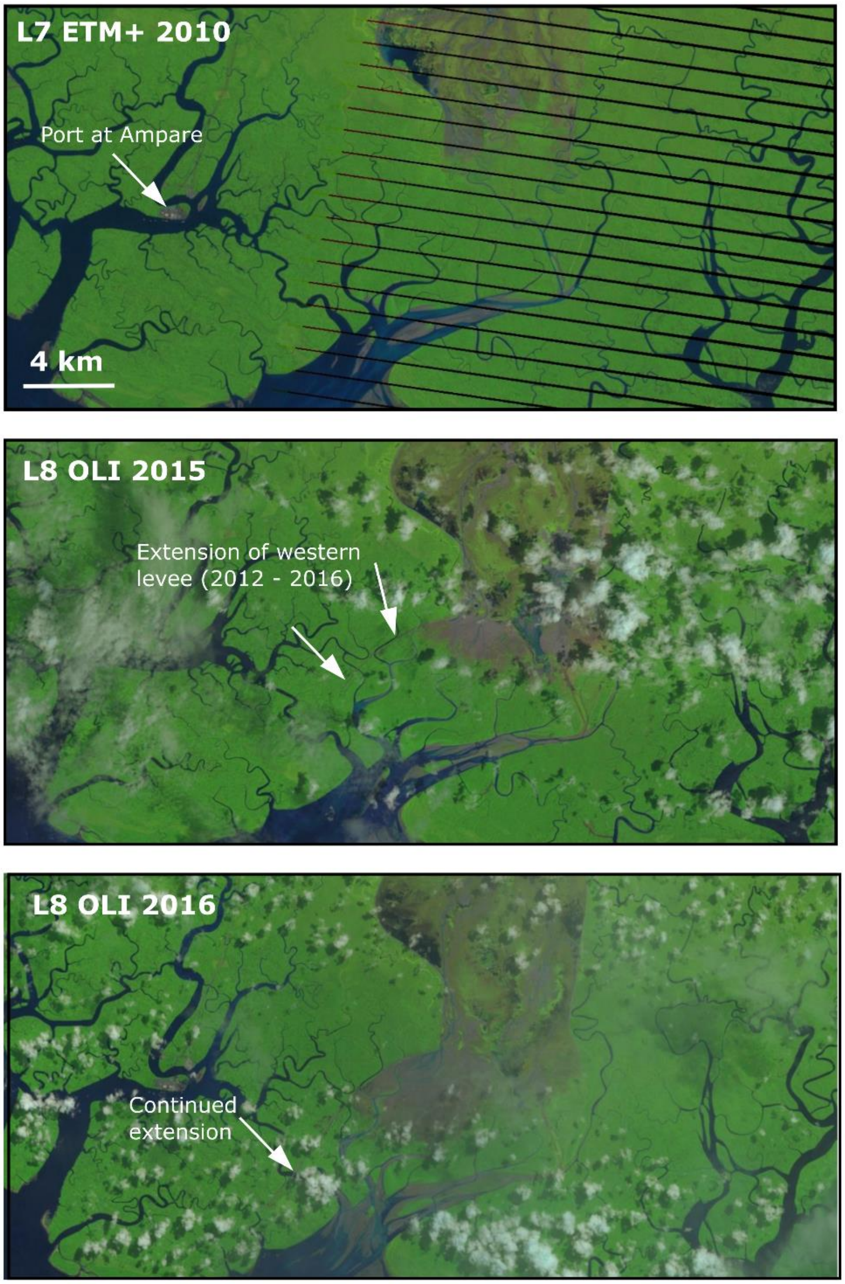

Figure S4: Recent Landsat 7 and Landsat 8 true color imagery shows substantial ADA expansion along the newly-extended western levee. The levee was extended because tailings were threatening the port at Ampare. Figure assembled in Inkscape v0.91 (www.inkscape.org) from Landsat surface reflectance imagery available from the U.S. Geological Survey.

Video V1: Modeled, per-pixel, cloud-free, annual change in NDVI (0-1 scale) from 1987 to 2014 based on the Noise Insensitive Trajectory Algorithm. Video created in Matlab 2015 using Landsat surface reflectance imagery available from the U.S. Geological Survey.

References for supplemental materials:

Lobo, F. L., Costa, M. P. F., & Novo, E. M. L. M. (2014). Time-series analysis of Landsat-MSS/TM/OLI images over Amazonian waters impacted by gold mining activities. *Remote Sensing of Environment*, 157, 170–184.
